# Integrative analysis of histomorphology, transcriptome and whole genome resequencing identified *DIO2* gene as a crucial gene for the protuberant knob located on forehead in geese

**DOI:** 10.1186/s12864-021-07822-9

**Published:** 2021-06-30

**Authors:** Yan Deng, Shenqiang Hu, Chenglong Luo, Qingyuan Ouyang, Li Li, Jiaming Ma, Zhenping Lin, Junpeng Chen, Hehe Liu, Jiwei Hu, Guohong Chen, Dingming Shu, Yuxuan Pan, Bo Hu, Hua He, Hao Qu, Jiwen Wang

**Affiliations:** 1 Farm Animal Genetic Resources Exploration and Innovation Key Laboratory of Sichuan Province, Sichuan Agricultural University, Sichuan 611130 Chengdu, China; 2 The Institute of Animal Science, Guangdong Academy of Agricultural Sciences, Guangdong 510640 Guangzhou, China; 3 The Baisha Livestock and Poultry Original Species Research Institute, Guangdong 515000 Shantou, China; 4 Jiangsu Key Laboratory for Animal Genetic, Breeding and Molecular Design, Yangzhou University, Jiangsu 225009 Yangzhou, China

**Keywords:** Cranial appendage, Knob, Histomorphology, Genetic mechanism, DIO2, Goose

## Abstract

**Background:**

During domestication, remarkable changes in behavior, morphology, physiology and production performance have taken place in farm animals. As one of the most economically important poultry, goose owns a unique appearance characteristic called knob, which is located at the base of the upper bill. However, neither the histomorphology nor the genetic mechanism of the knob phenotype has been revealed in geese.

**Results:**

In the present study, integrated radiographic, histological, transcriptomic and genomic analyses revealed the histomorphological characteristics and genetic mechanism of goose knob. The knob skin was developed, and radiographic results demonstrated that the knob bone was obviously protuberant and pneumatized. Histologically, there were major differences in structures in both the knob skin and bone between geese owing knob (namely knob-geese) and those devoid of knob (namely non-knob geese). Through transcriptome analysis, 592 and 952 genes differentially expressed in knob skin and bone, and significantly enriched in PPAR and Calcium pathways in knob skin and bone, respectively, which revealed the molecular mechanisms of histomorphological differences of the knob between knob- and non-knob geese. Furthermore, integrated transcriptomic and genomic analysis contributed to the identification of 17 and 21 candidate genes associated with the knob formation in the skin and bone, respectively. Of them, *DIO2* gene could play a pivotal role in determining the knob phenotype in geese. Because a non-synonymous mutation (c.642,923 G > A, P265L) changed DIO2 protein secondary structure in knob geese, and Sanger sequencing further showed that the AA genotype was identified in the population of knob geese, and was prevalent in a crossing population which was artificially selected for 10 generations.

**Conclusions:**

This study was the first to uncover the knob histomorphological characteristics and genetic mechanism in geese, and *DIO2* was identified as the crucial gene associated with the knob phenotype. These data not only expand and enrich our knowledge on the molecular mechanisms underlying the formation of head appendages in both mammalian and avian species, but also have important theoretical and practical significance for goose breeding.

**Supplementary Information:**

The online version contains supplementary material available at 10.1186/s12864-021-07822-9.

## Background

Goose is a migratory bird and worldwide distributed, the wild progenitors (i.e., Greylag Goose (*Anser anser*) and Swan Goose (*Anser cygnoides*)) of the domestic geese of the world are predicted to share a common ancestor about 3.4 Mya [1]. Upon a long-term period of domestication and hybridization, a diverse set of domestic geese breeds have been produced and are generally divided into European and Chinese geese according to their evolutionary history and geographical distribution [2]. These breeds vary remarkably in their behavior, morphology, growth and development, as well as reproduction and endocrinology. For instance, knob, locating at the base of the upper bill and known as a unique appearance characteristic of domestic geese, merely exists in domestic breeds derived from *Anser cygnoides* rather than *Anser anser*. Moreover, knob is also absent in the wild progenitors of the domestic geese. According to the experienced breeders, the individual with a large knob appears to have a higher social rank, and sex-dependent differences in the knob size have been observed in a number of goose breeds, such as Lion head goose, Sichuan white goose, and Tianfu Meat goose, a crossing population. However, neither the histomorphological characteristics nor the formation mechanism of the knob phenotype has been revealed in domestic geese.

Compared to the situation in geese, studies focusing on the histomorphology and genetic mechanism of resembled cranial appendages on the head have been extensively conducted in both mammalian species and other avian species. In view of appearance, the head cranial appendages of mammals, such as horns in *Cervidae* and *Bovidae*, were paired and symmetric. They were located on the frontal bones and were covered by skin and connective tissues or were keratinized, and the horn bony core was pneumatized [3]. In birds, the head cranial appendages mainly included comb and crest. Comb was a fleshy protuberance located at the top of the head in chickens [4], while crest was either a bony protuberance located in the anterodorsal region of the skull in crested chickens [5] or a cushion composed of fat and connective tissue in the parietal part of the skull in crested ducks [6], or the feathers grew toward the top of the head instead of down the neck in pigeons [7]. Although the horns in both *Cervidae* and *Bovidae* have been demonstrated to be originated from neural crest stem cells [8], their genetic basis is different. Specifically, the horn’s morphology in *Bovidae* was determined by a single autosomal locus (i.e., *Horns*) and was most likely regulated by the relaxin family peptide receptor 2 (*RXFP2*) gene, known as a candidate gene for *Horns*, in sheep and cattle [9–11]. In contrast, the Wnt signaling pathway was evidenced to play a key role in stimulating the differentiation of antler stem cells and promoting chondrogenesis and osteogenesis during the development of antler [12]. Compared with mammals, the comb and crest in birds were reported to be caused by either genomic structure variations or mutations in the *cis*-acting regulatory elements, eventually leading to the ectopic expression of some key transcription factors [13–15]. These results altogether suggested that the formation mechanisms of the head cranial appendages are different between mammalian and avian species. More importantly, a number of studies have indicated that the morphology of the head cranial appendage was tightly related to both the physiology and production performance in farm animals [16–18]. As one of the most economically important poultry, it is of great importance to investigate both the histomorphological characteristics and genetic mechanism of the knob in geese.

A growing number of studies have been conducted to reveal the genetic basis of multiple phenotypic traits through integrated analysis of genome and transcriptome sequencing data [19, 20]. Moreover, the publication of goose reference genome makes it possible to reveal the genetic basis of goose phenotypic traits at the genomic level [21]. Therefore, the present study aimed to compare the histomorphological characteristics of both the knob skin and bone between geese owing knob (namely knob-geese) and those devoid of knob (namely non-knob geese) and to identify the crucial genes associated with the formation of knob in geese. These results would provide a theoretical foundation for the further revelation of genetic basis of knob in geese. To our knowledge, this study represents the first to develop research on the knob phenotype in goose, and it will contribute to a better understanding of the genotype-phenotype relationships in domestic animals and efficiently facilitate goose breeding.

## Results

### Histomorphological differences in the knob skin between knob- and non-knob geese

**Fig. 1:**
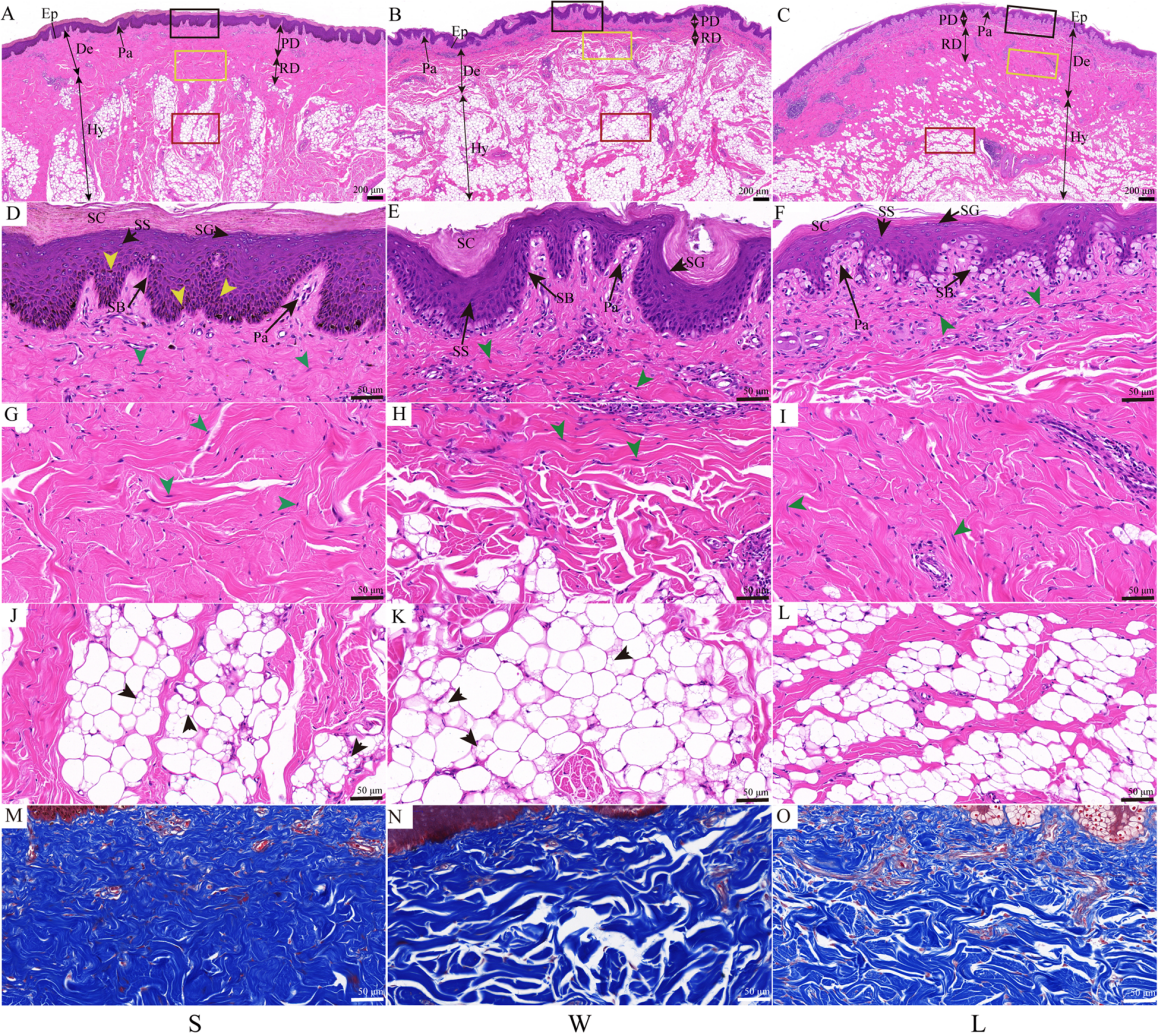
Histology of skin located on knob between knob- and non-knob geese. **A-C** Low-magnification photomicrograph of HE-stained skin. Ep, epidermis; De, dermis; Hy, hypodermis; Pa, papillary; PD, papillary dermis; RD, reticular dermis; the black, yellow and red rectangles represented **D-F**, **G-I**, and **J-L** at high-magnification, respectively; Scale bar: 200 μm. **D-L** High-magnification photomicrograph of HE-stained skin. SC, stratum corneum; SG, stratum granulosum; SS, stratum spinosum; SB, stratum basal; yellow arrowhead, pigment particles; green arrowhead, fibroblast; black arrowhead, locular adipocytes. Scale bar: 50 μm. **M-O** High-magnification photomicrograph of Masson-stained skin. Scale bar: 50 μm. S, Lion head goose; W, Sichuan White goose; **L**, Landes goose. **S** and **W** are with knob, **L** is devoid of knob. The first column represented **S**, the second column represented **W**, and the third column represented **L**

The H&E staining results showed that the histological structure of the knob skin in both knob- (Lion head goose and Sichuan White goose, S and W) and non-knob geese (Landes goose, L) was composed of epidermis, dermis and subcutaneous connective tissue (Fig. 1A-C). Among them, the epidermis was further divided into stratum corneum (SC), stratum granulosum (SG), stratum spinosum (SS), stratum basal (SB) (Fig. 1D - F). The dermis was further classified into superficial papillary dermis (Fig. 1D - F) and deeper reticular dermis (Fig. 1G - I). Subcutaneous connective tissue was primarily composed of tightly packed, large unilocular adipocytes and a few fibroblasts (Fig. 1J - L).

In knob geese, however, the thicknesses of epidermis, SC, as well as the height of papillary layer in dermis significantly increased (Additional file 2: Figure S2 (A, B, E), *p* < 0.05). The cells located in SB were columnar or cuboid and were arranged neatly (Fig. 1D, E), and numerous smaller locular adipocytes were scattered in the large locular adipocytes in knob geese (Fig. 1J, K). In non-knob geese, the cells located in SB were arranged irregularly and the cytoplasm was shallow (Fig. 1F), as well as a few smaller locular adipocytes were observed in the large locular adipocytes (Fig. 1L). In addition, the thicknesses of papillary and reticular dermis significantly increased in S than that in W and L (Additional file 2: Figure S2 (C - D), *p* < 0.05), while that were not significant differences between W and L (Additional file 2: Figure S2 (C - D), *p* > 0.05). Meanwhile, the content of collagen in dermis was the highest in S (Fig. 1M-O, Additional file 2: Figure S2F, *p* < 0.05). The density of adipocytes was significantly higher in W (Additional file 2: Figure S2G, *p* < 0.05), while there was not significant difference between S and L (Additional file 2: Figure S2G, *p* > 0.05). Taken together, these results suggested that the histomorphology of the knob skin between knob- and non-knob geese was significant difference in the thicknesses of epidermis, dermis and in cell morphology located in SB and subcutaneous connective tissue.

### Histomorphological differences in the knob bone between knob- and non-knob geese

**Fig. 2:**
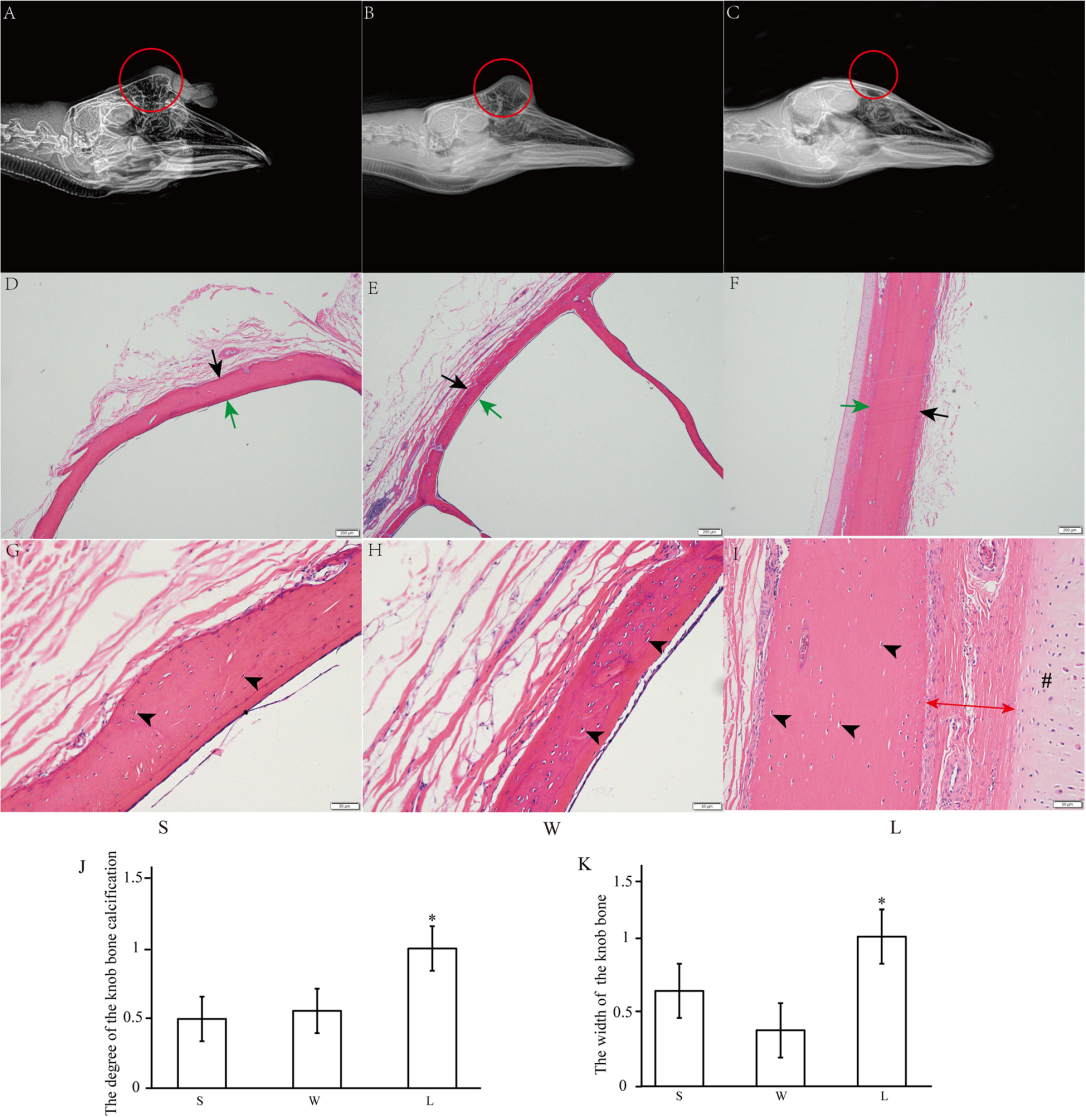
Histomorphology of bone located in knob between knob- and non-knob geese. **A-C** Right latero-lateral radiograph images of heads of different geese breeds. The position of bone protuberances was red circled. **D-F** Low-magnification photomicrograph of HE-stained bone. Black arrow, external periosteum; green arrow, internal periosteum. Scale bar: 200 μm. **G-I** High-magnification photomicrograph of HE-stained bone. Black arrowhead, osteocyte; double-arrow, the connective tissue; #, cartilage tissue. Scale bar: 50 μm. **J-K** The calcification degree and width of the knob bone in different geese breeds. The data of **L** was used for normalization of the data of both **S** and **W**, and the data was displayed as multiples of changes. * represented the statistically significance. *P* < 0.05. S, Lion head goose; W, Sichuan White goose; L, Landes goose. S and W are with knob, L is devoid of knob

As shown in Fig. 2, in knob geese (S and W), the bone located in the knob was obviously protruding, and the bony core of the knob bone was pneumatized (Fig. 2A-B). In non-knob geese (L), however, the bone was not protruding (Fig. 2C). The degree of bone calcification was significantly decreased in knob geese than that in non-knob geese (Fig. 2J, *p* < 0.05). The H&E staining results showed that the bone of both knob- and non-knob geese had mature bone matrix containing osteocyte, and bone matrix was surrounded by internal and external periosteum interstitial tissue (Fig. 2G-I). Histological parameters analysis showed that the thickness of the bone was significantly thicker in non-knob geese than that in knob geese (Fig. 2D-F and K, *p* < 0.05). In addition, the connective tissue and cartilage were also observed close to the bone matrix in non-knob geese (Fig. 2I). Thus, histomorphological analysis of the knob bone showed that the knob bone was protruding and pneumatized, and the degree of the knob bone calcification and the thickness of the bone significantly decreased in knob geese.

### Comparative transcriptome analysis of the knob skin and bone between knob- and non-knob geese

**Fig. 3:**
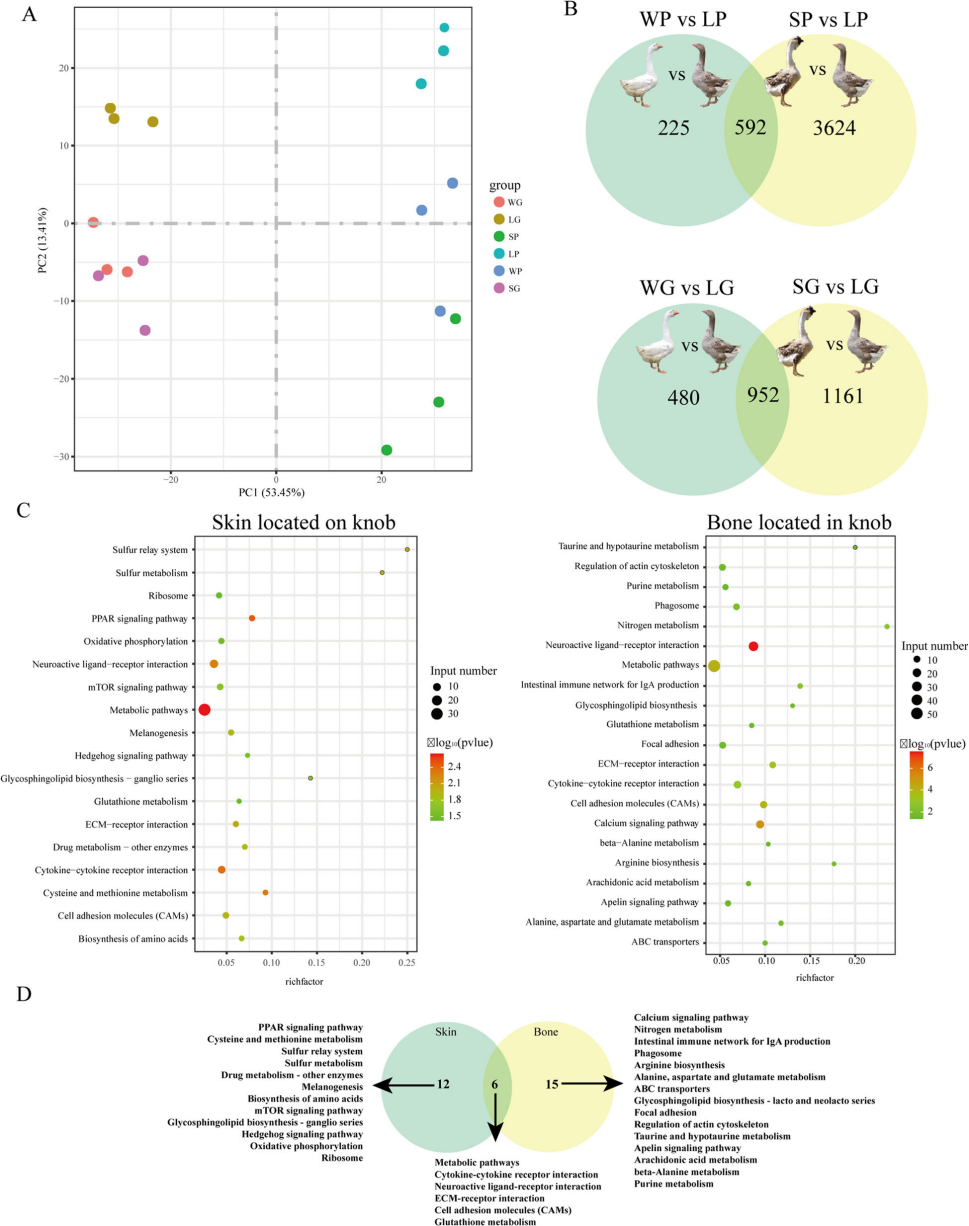
Transcriptomic analysis of both skin and bone located in knob between knob- and non-knob geese. **A **Principal component analysis of the knob skin and bone samples. **B** The number of differentially expressed genes (DEGs) in the knob skin and bone between knob- and non-knob geese, respectively. **C** Functional enrichment of DEGs in the knob skin and bone between knob- and non-knob geese, respectively. **D** The enrichment pathways in the knob skin were intersected with these in the knob bone. WP, skin located on knob in Sichuan White goose; SP, skin located on knob in Lion head goose; LP, skin located on knob in Landes goose; WG, bone located in knob in Sichuan White goose; SG, bone located in knob in Lion head goose; LG, bone located in knob in Landes goose. W and S represented the knob group, L represented the non-knob group

To investigate the molecular mechanism of the knob phenotype, comparative analysis of transcriptome for the knob skin and bone between knob- and non-knob geese was performed. After removing low-quality reads and adaptor sequences, a range of 45,127,094 to 66,029,052 clean reads were generated by all libraries, and Q20 ratio, Q30 ratio varied from 96.19 to 97.37 %, 90.43 to 92.99 %, respectively (Additional file 1: Table S3). The mapping rates of unique reads were from 68.55 to 79.66 % among all libraries (Additional file 1: Table S4). Principal component analysis (PCA) divided the knob skin and bone into separate clusters (Fig. 3 A). Among them, the skin of Lion head goose (SP), the skin of Sichuan White goose (WP) and the skin of Landes goose (LP) were further divided into separate clusters (Additional file 2: Figure S3A), while the bone of Lion head goose (SG) and the bone of Sichuan White goose (WG) were divided into a cluster (Additional file 2: Figure S3B). Analysis of differentially expressed genes (DEGs) showed that 981, 4862 and 2145 DEGs were found in WP vs. LP, SP vs. LP, WP vs. SP, respectively, and 1711, 2535 and 778 DEGs were observed in WG vs. LG, SG vs. LG, WG vs. SG, respectively (Additional file 1: Table S5). When the DEGs in WP vs. LP were overlapped with that in SP vs. LP, 592 DEGs were identified in knob skin between knob- and non-knob geese. When the DEGs in WG vs. LG were overlapped with that in SG vs. LG, 952 DEGs were identified in knob bone between knob- and non-knob geese (Fig. 3B).

Analysis of functional enrichment showed that the DEGs in the knob skin were significantly enriched in Metabolic pathways, PPAR signaling pathway, Cytokine-cytokine receptor interaction, Neuroactive ligand-receptor interaction, ECM-receptor interaction, Biosynthesis of amino acids (Fig. 3C). In the knob bone, the DEGs were significantly enriched in Neuroactive ligand-receptor interaction, Calcium signaling pathway, Metabolic pathways, Cell adhesion molecules (CAMs), ECM-receptor interaction, Cytokine-cytokine receptor interaction (Fig. 3C). Among these pathways, Metabolic pathways, Cytokine-cytokine receptor interaction, Neuroactive ligand-receptor interaction, ECM-receptor interaction, Cell adhesion molecules (CAMs) and Glutathione metabolism were shared in the knob skin and bone (Fig. 3D). Combined with the results of histomorphology, PPAR signaling and Calcium signaling pathways might play key roles in the knob skin and bone between knob- and non-knob geese, respectively (Fig. 3D). The DEGs enriched in these pathways were listed in Table 1.

**Table 1:** Differentially expressed genes enriched in PPAR signaling pathway, Calcium signaling pathway and ECM-receptor interaction (*p* < 0.05)

**Pathways**	**DEGs**	**WPvsLP_log2FoldChange**	**SPvsLP_log2FoldChange**
PPAR signaling pathway	*FABP7*	1.47	2.07
	*LPL*	2.34	2.58
	*FABP3*	3.31	4.39
	*UBB*	1.51	1.56
	*ACSBG1*	1.32	1.98
**Pathways**	**DEGs**	**WGvsLG_log2FoldChange**	**SGvsLG_log2FoldChange**
Calcium signaling pathway	*CD38*	-2.00	-2.01
	*CACNA1H*	-1.79	-1.92
	*NOS2*	2.96	1.43
	*ATP2B2*	2.76	2.92
	*CAMK1G*	-2.22	-1.62
	*CACNA1I*	3.39	3.18
	*NOS1*	-3.30	-3.84
	*RYR2*	-2.06	-1.56
	*HTR4*	-2.83	-2.30
	*AVPR1A*	-2.01	-1.14
	*PTAFR*	1.66	1.30
	*HTR2C*	-2.55	-2.23
	*HRH1*	-1.78	-1.24
	*P2RX1*	-2.56	-1.63
	*GRIN1*	-6.62	-4.08
	*ADCY3*	-2.30	-3.24
	*GRM1*	6.21	5.55
**Pathways**	**DEGs**	**WPvsLP_log2FoldChange**	**SPvsLP_log2FoldChange**
ECM-receptor interaction	*HMMR*	-2.26	-2.19
	*VTN*	4.30	3.24
**Pathways**	**DEGs**	**WGvsLG_log2FoldChange**	**SGvsLG_log2FoldChange**
ECM-receptor interaction	*HMMR*	-3.00	-2.77
	*VTN*	2.44	2.03

*Note*: *DEGs* differently expressed genes, *WP* skin located on knob in Sichuan White goose, *SP* skin located on knob in Lion head goose, *LP* skin located on knob in Landes goose, *WG* bone located in knob in Sichuan White goose, *SG* bone located in knob in Lion head goose, *LG* bone located in knob in Landes goose

### Identification of genetic variants between knob- and non-knob geese through whole genome re-sequencing analysis

**Fig. 4:**
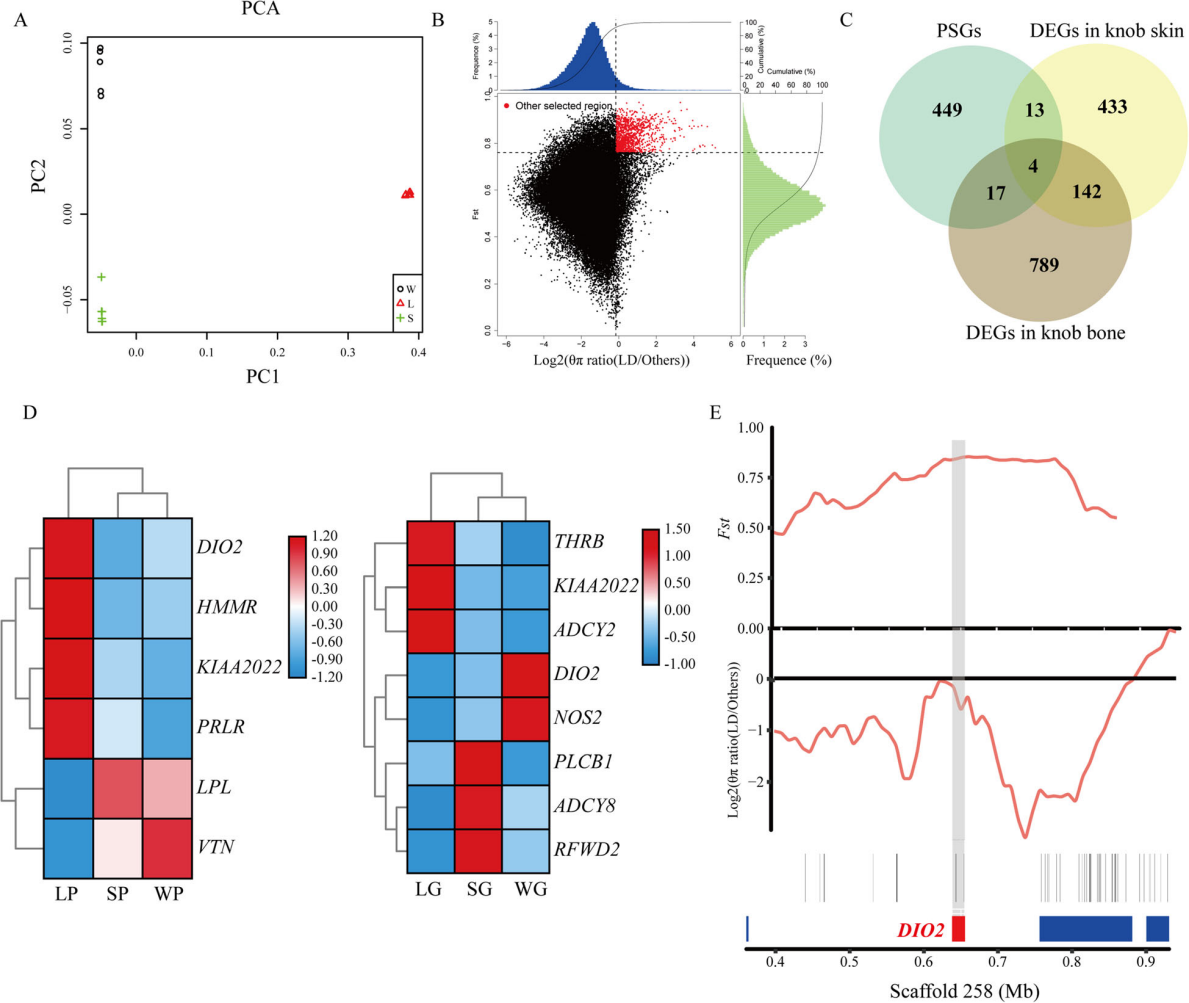
Whole genome-resequencing analysis and identification of candidate genes associated with knob phenotype. **A** Principal component analysis among 3 different geese breeds. W, Sichuan White goose; L, Landes goose; S, Lion head goose. **B** Distribution of *Fst* and log2(θ_π_ ratio (LD/Others)) values (indicated by green and blue colors, respectively) calculated in sliding 50-kb windows with 10-kb steps. The data pointed in red were genomic regions under strong selection. **C** PSGs were intersected with the DEGs in the knob skin and bone. PSGs, candidate genes under positive selection; DEGs, differentially expressed genes. **D** Heatmap of 12 crucial genes associated with selected genes and major pathways. LP, skin located on knob of Landes goose; SP, skin located on knob of Lion head goose; WP, skin located on knob of Sichuan White goose; LG, bone located in knob of Landes goose; SG, bone located in knob of Lion head goose; WG, bone located in knob of Sichuan White goose. **E **Genomic region with strong selective sweep signals on scaffold 258 of goose. The values of *Fst* and log2(θπ ratio) were plotted. The shade represented the position of *DIO2* gene in this scaffold

To further investigate the genetic diversity of the knob at the genomic level, whole genome re-sequencing was developed in three domestic geese breeds. Results showed that whole genome re-sequencing of 18 individuals generated a total of 5.11 billion clean reads with an average depth of ~ 31.43 x. These reads were aligned to the *Anser. Cygnoides* reference genome with an average mapping rate of 94.82 % that covered 99.46 % of the reference genome, indicating that the data had high quality and could be used for further in-depth analysis (Additional file 1: Table S6). Next, a total of 21,593,064 SNPs was analyzed across 18 individuals (Additional file 1: Table S7). Most (≥ 93.8 %) of the SNPs were presented in intergenic and intronic regions. The remaining SNPs were located in upstream, downstream of open reading frame and exonic region. PCA based on the SNPs showed that the population of L and other two geese breeds were separated into two clusters (Fig. 4A). Top 5 % of both *Fst* and log2(*θ*_*π*_ ratio (_LD/Others_)) were used as the standard of selection, 120 candidate regions under positive selection between knob- (S and W) and non-knob geese (L) were identified and 483 candidate genes (PSGs) under positive selection harbored in these regions were annotated (Fig. 4B, Additional file 1: Table S8).

### Identification of crucial candidate genes associated with goose knob phenotype through integrated transcriptomic and genomic analysis

To reveal the crucial candidate genes associated with the knob phenotype, 483 PSGs were intersected with DEGs, which were identified in skin and bone located in the knob between knob- and non-knob geese, respectively. Finally, 17 and 21 candidate genes were identified in the knob skin and bone, respectively, and four genes (iodothyronine deiodinase 2 (*DIO2*), neurite extension and migration factor (*KIAA2022*), ADAM metallopeptidase with thrombospondin type 1 motif 3 (*ADAMTS3*), transient receptor potential cation channel subfamily C member 6 (*TRPC6*)) were shared in both knob skin and bone (Fig. 4C, Additional file 1: Table S9). Compared to non-knob geese, genes including epoxide hydrolase 4 (*EPHX4*) and goosecoid homeobox (*GSC*) in the knob skin and trans-golgi network vesicle protein 23 homolog A (*TVP23A*), class II major histocompatibility complex transactivator (*CIITA*), F-box and leucine rich repeat protein 17 (*FBXL17*), COP1 E3 ubiquitin ligase (*RFWD2*), calcium voltage-gated channel subunit alpha1 I (*CACNA1I*), *DIO2* in the knob bone were significantly upregulated in knob geese. Meanwhile, genes including *KIAA2022*, *ADAMTS3*, *DIO2*, *TRPC6*, teneurin transmembrane protein 1 (*TENM1*), *LOC106047464*, fibrillin 2 (*FBN2*) etc. in the knob skin and *KIAA2022*, *ADAMTS3*, *TRPC6*, regulator of G protein signaling 7 (*RGS7*), regulating synaptic membrane exocytosis 1 (*RIMS1*), phospholipase A2 group IVF (*PLA2G4F*), *LOC106042972*, ABI family member 3 binding protein (*ABI3BP*) etc. in the knob bone were significantly downregulated in knob geese (Additional file 1: Table S9). Based on RNA-seq and whole genome re-sequencing analyses, several genes involved in PPAR signaling pathway, ECM-receptor interaction and Calcium signaling pathway as well as candidate genes were selected for qRT-PCR validation. As shown in Additional file 2: Figure S4, expression of all these selected genes showed changes in the same direction with that observed using RNA-seq, indicating the results of RNA-seq were reliable. In addition, clusters analysis of expression of these selected genes showed that in the knob skin, the expression patterns of *DIO2*, hyaluronan mediated motility receptor (*HMMR*), *KIAA2022* and prolactin receptor (*PRLR*) were clustered and their expressions were downregulated in knob geese, whereas the expression pattern of lipoprotein lipase (*LPL*) was clustered with that of vitronectin (*VTN*) and their expressions were upregulated in knob geese (Fig. 4D). Similarly, in the knob bone, the expression patterns of thyroid hormone receptor beta (*THRB*), *KIAA2022* and adenylate cyclase 2 (*ADCY2*) were clustered and their expressions were downregulated in knob geese. In addition, the expression patterns of *DIO2* and nitric oxide synthase 2 (*NOS2*), that of phospholipase C beta 1 (*PLCB1*), adenylate cyclase 8 (*ADCY8*) and *RFWD2* were clustered, respectively, and their expressions were upregulated in knob geese (Fig. 4D).

### A non-synonymous mutation in *DIO2* gene was strongly associated with the knob phenotype in geese

**Fig. 5:**
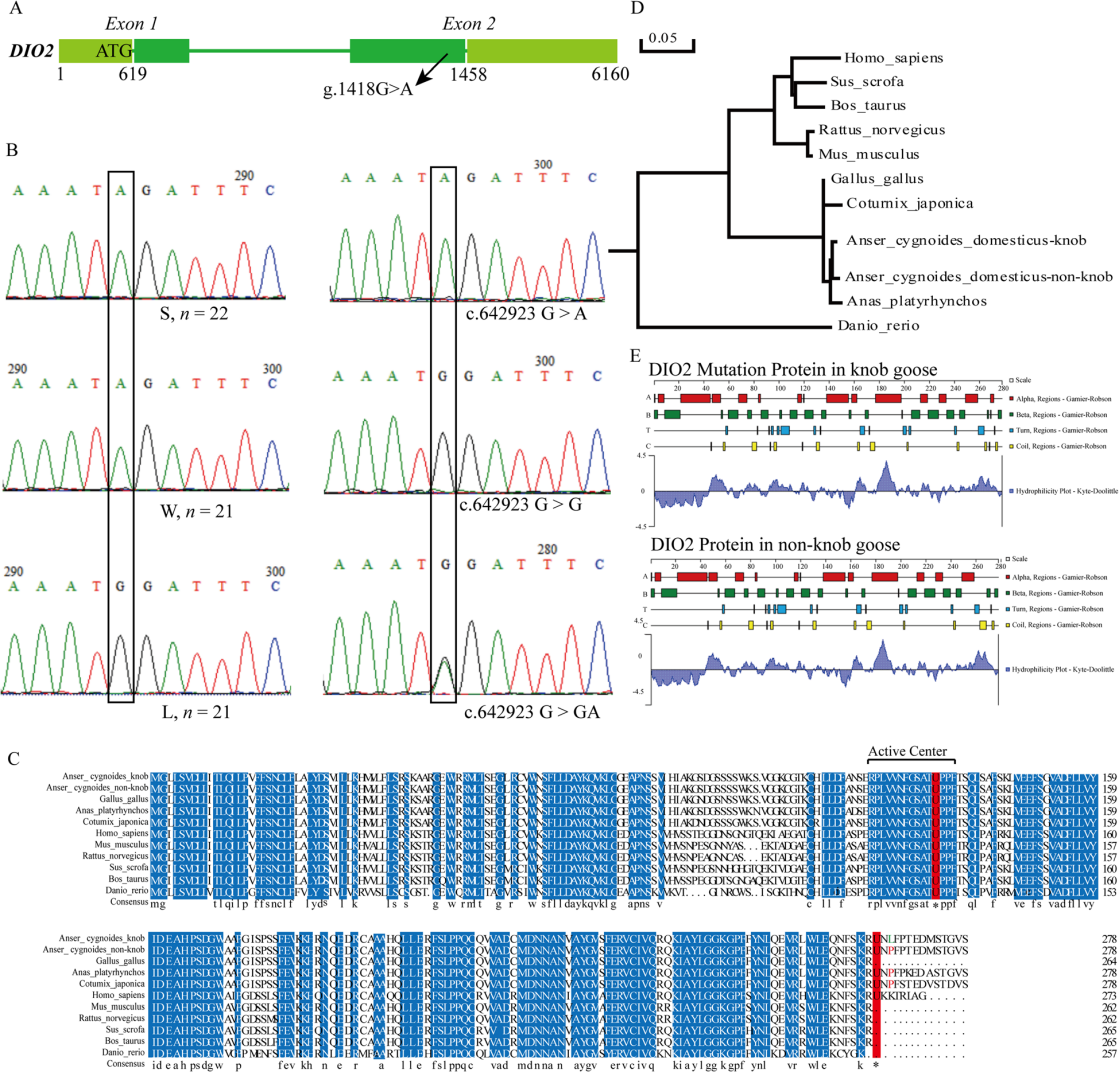
Validation of a non-synonymous mutation in *DIO2* gene and bioinformation analysis of DIO2 protein. **A** The position of the non-synonymous mutation in *DIO2* gene. **B** Validation of the non-synonymous mutation in *DIO2* gene in the population of S, W and L (left) and population of Tianfu meat geese breed (right). Black rectangle represented the mutant base in the population of S, W and L and population of Tianfu meat geese breed. S, Lion head goose; W, Sichuan White goose; L, Landes goose. “*n*” represented the population number of each breeds. **C** Homology comparison of DIO2 protein among different species. Consistent amino acids were shown in blue among different species. The Sec residues were shown in highlight red. Primary amino acid was shown in red, and mutant amino acid was shown in green. The number in the right represented the position of amino acid. **D** Phylogenetic tree analysis of *DIO2* among different species. **E** Analysis of the secondary structure of DIO2 protein in knob- and non-knob geese

Among these crucial candidate genes associated with the knob phenotype, the expression of *DIO2* was downregulated in the knob skin and upregulated in the knob bone in knob geese. Population diversity analysis showed that the whole genome population differentiation value of *DIO2* was high (Fig. 4E, Additional file 1: Table S9, *Fst* = 0.8095). Genetic variants showed that knob geese showed a G/A non-synonymous mutation (P265L) in the coding region of *DIO2* compared to non-knob geese (L, Fig. 5 A). Further protein sequence analysis of *DIO2* gene showed that the non-synonymous mutation was not located in the active center of the DIO2 enzyme and the amino acid sequence of the non-synonymous mutation was the same among non-knob geese, duck (*Anas_platyrhynchos*) and quail (*Coturnix_japonica*) (Fig. 5 C). While the non-synonymous mutation and the following sequence were missing in chicken and mammals (Fig. 5 C). Phylogenetic analysis showed that DIO2 in knob geese was also clustered with that in non-knob geese (Fig. 5D). The protein secondary structure prediction of DIO2 showed that the proportion of α helix decreased by 3.25 %, that of extended strand increased by 1.08 % and that of random coil increased by 2.17 % in knob geese (Fig. 5E). Furthermore, the non-synonymous mutation has been verified by Sanger sequencing among the population of S, W and L as well as the population of Tianfu meat geese breed (Fig. 5B). In the population of S, W and L, all of non-knob geese (L) individuals showed GG genotype, whereas all of knob geese (S and W) individuals showed AA genotype. In the population of Tianfu meat geese breed, three genotypes (GG, GA and AA) and two alleles (G and A) were identified. Knob geese individuals with AA genotypes were more prevalent than non-knob geese individuals with GG genotype, and the frequency of the A allele was higher than that of the G allele (Table 2). All of these results indicated that *DIO2* might be the crucial gene for the formation of knob in geese.

**Table 2:** Genotypes and allele frequencies of the mutation locus of *DIO2* gene in Tianfu meat geese breed

Position	Genotype frequencies			Allelic frequencies		*P*-value
	AA	GA	GG	A	G	
c.642,923 G > A	0.32(96)	0.47(143)	0.21(65)	0.55	0.45	0.39

## Discussion

Compared to other cranial appendages, the histomorphology and genetic mechanism of the knob remains poorly understood in geese. In the present study, the radiographic, histological, transcriptomic, and genomic characteristics of both the knob skin and bone were comprehensively examined and compared among three domestic geese breeds showing distinct knob phenotypes. Histomorphological analysis showed that goose knob was characterized by thickened skin and protruding bone. Although the structure of the knob skin was similar among three examined geese breeds, there were major differences in the thicknesses of epidermis and dermis, the content of collagen and the density of adipocytes in the knob skin between knob- and non-knob geese. Similarly, the structure of the knob bone was also similar among three examined geese breeds, but the thickness and calcification of the knob bone differed significantly between knob- and non-knob geese. These results indicated that there were major differences in the histological structure of the knob between knob- and non-knob geese, and the knob phenotype was postulated to be resulted from the synergistical contributions of both the skin and bone.

**Fig. 6:**
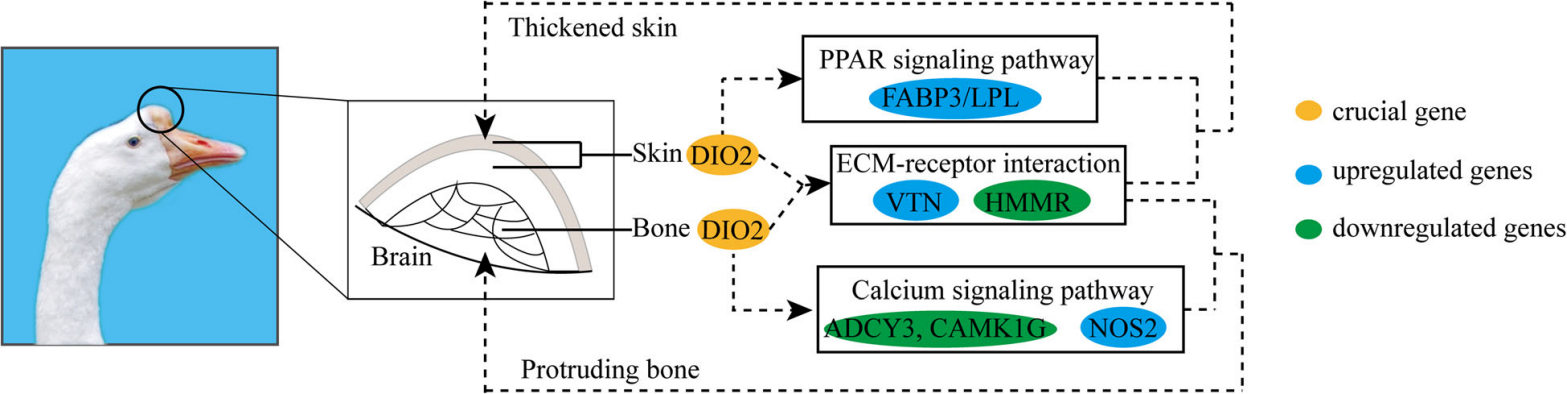
Diagram of the knob formation mediated by *DIO2* in knob geese. “⇢” indicated the possible pathways

At the transcriptomic level, a total of 592 and 952 DEGs were identified in the knob skin and bone between knob- and non-knob geese, respectively. Subsequent functional enrichment analysis showed that the Calcium signaling, Apelin signaling and Metabolic pathways were specifically enriched in the knob bone, while the PPAR, mTOR and Hedgehog signaling pathways were specifically enriched in the knob skin. It has been well demonstrated that the above signaling pathways played important roles in generating cellular signals required for normal physiology [22] and regulating fundamental physiological processes including protein synthesis to autophagy and embryonic development [23, 24]. Combined with the histological results, we speculated that the PPAR signaling and Calcium signaling pathways in either the knob skin or bone could play crucial roles in determining the knob phenotype. PPAR signaling pathway played an important role in fatty acid oxidation and lipid synthesis [22], and the expression of genes associated with fatty acid synthesis, such as fatty acid binding protein 3/7 (*FABP3/7*) [25] and *LPL* [26], were upregulated in the knob skin in knob geese. Calcium was an intracellular messenger to mediate the cell proliferation and apoptosis, growth, and cell division [27–29], and the expression of genes associated with calcium ion homeostasis and signaling, such as glutamate ionotropic receptor NMDA type subunit 1 (*GRIN1*) and calcium/calmodulin dependent protein kinase IG (*CAMK1G*) [30], were downregulated in the knob bone in knob geese. These results suggested that the thickened skin and protruding bone in knob geese could be associated with the fatty acid synthesis and the bone calcification, which was mediated by the PPAR and Calcium signaling pathway, respectively. Conversely, with the exemption of the specific signaling pathways in the knob skin and bone, metabolic pathways, cytokine-cytokine receptor interaction, neuroactive ligand-receptor interaction, ECM-receptor interaction and cell adhesion molecules were shared in both knob skin and bone. These signaling pathways were known to regulate different cell biological processes, such as the morphogenesis and the maintenance of structure and function, cell proliferation and survival [31], and skin development [32, 33]. Among them, extracellular matrix (ECM) played a necessary role in the morphogenesis of tissue and organ and in the maintenance of cell and tissue structure and function [31]. A recent study showed that ECM provided structural support for adipocytes and regulated adipogenesis, which made a contribution to the deposition of fat [34]. As for genes involved in the ECM-receptor interaction pathway, expression of *HMMR* was downregulated while that of *VTN* was upregulated in the knob skin and bone in knob geese. It was shown that downregulation of *HMMR* expression promoted pre-adipocyte differentiation, while upregulation of *HMMR* expression suppressed the expression levels of peroxisome proliferator activated receptor gamma (*PPARγ*), C/EBP and downstream target genes in adipocytes and activated extracellular signal-regulated kinase1/2 (ERK1/2) to promote the proliferation of osteoblastic cells [35–37]. *VTN* was a multifunctional plasma glycoprotein that played a role in cell adhesion and cell migration through pericellular proteolysis and growth factor signaling [38]. These results indicated that the thickened skin and the protruding bone of the knob in knob geese might be simultaneously regulated by the ECM-receptor interaction pathway. Taken together, the knob formation in geese was the result of both the synergistical contributions of skin and bone through the ECM-receptor interaction pathway and the independent contribution of skin and bone through the PPAR and Calcium signaling pathways, respectively (Fig. 6).

Furthermore, integrated analysis of whole genome re-sequencing and transcriptome sequencing data identified 17 and 21 candidate genes associated with the knob phenotype in the knob skin and bone, respectively. Among these genes, *DIO2* was simultaneously differentially expressed in both the knob skin and bone between knob- and non-knob geese and was strongly selected at the genomic level. *DIO2* was the primary enzyme of metabolic regulation of thyroid hormones (THs) and catalyzed thyroxine (T4) to triiodothyronine (T3) [39]. A previous study has shown that the expression level of *DIO2* was negatively related to the fatty acid synthesis, oxidation and mitochondrial function [40]. In the present study, the expression level of *DIO2* was negatively related to that of *LPL* involved in fatty acid synthesis, indicating that *DIO2* could affect the formation of knob skin in knob geese through mediating fatty acid synthesis and metabolism. Analysis of genetic variation showed that a non-synonymous mutation was identified in the coding region of *DIO2* in knob geese, and the amino acid was not located in the conserved deiodinase catalytic domain. Previous studies showed that although a missense mutation (T92A) in *DIO2* was not phylogenetically conserved [41], the missense mutation in *DIO2* was strongly associated with insulin resistance [41, 42], thyroid stimulating hormone (TSH) level [43], reduced TSH-stimulated T3 release [44], decreased bone mineral density of femoral neck and higher bone turnover [45] in humans. These results demonstrated that a phylogenetically non-conservative missense mutation could make the alteration of physiological functions of *DIO2* gene. Furthermore, homology comparison analysis showed that the amino acid sequence of this mutation locus in *DIO2* was the same among non-knob geese, duck and quail, while that was missing in chicken and cattle. Significantly, there were no cranial appendages in duck and quail, while the comb in chickens and horns in cattle were located at the top of the head. Moreover, the non-synonymous mutation also significantly changed the protein secondary structures of *DIO2* gene, while the physiological functions of *DIO2* caused by the non-synonymous mutation need to be verified in the future. In addition, the knob geese showed complete mutation in this locus and the frequencies of AA genotype or A allele in knob geese were higher than that in non-knob geese. These results suggested that the non-synonymous mutation in *DIO2* could play a crucial role during the formation of knob in geese. All the mentioned results indicated that the non-synonymous mutation in *DIO2* might be a causal mutation to induce the knob formation by mediating the independent or synergistical contributions of the knob skin and bone in geese (Fig. 6). This regulatory mechanism was different from that of cranial appendages in mammals and other birds, and was species-specific, which might be related to differences in the evolution, adaptation and production performance among species.

## Conclusions

In conclusion, the present study was the first to systematically uncover the histomorphology and molecular mechanism of the goose knob. Histomorphological and transcriptomic analysis showed that histomorphological differences in the knob between knob- and non-knob geese were determined by the independent or synergistical contribution of the knob skin and bone mainly through the PPAR signaling, Calcium signaling and ECM-receptor interaction pathways. Furthermore, integrated analysis of transcriptome and whole genome re-sequencing data identified a mutation which resulted in a non-synonymous mutation within the *DIO2* gene, which ultimately changed the protein secondary structure, in knob geese. These data not only constitute a framework for understanding the genetic mechanism of the knob phenotype in goose, but also allow a comparison with that of the cranial appendages in both mammals and other birds.

## Methods

### Animals and sample preparation

A total of three domestic geese breeds were used in this study, including Landes goose (L), Lion head goose (S) and Sichuan White goose (W). For histomorphological analysis, six three-year-old male individuals from S, W and L were sampled. For transcriptomic analysis, additional three male individuals from S, W and L were sampled for RNA extraction. For whole genome re-sequencing analysis, blood of six individuals from each breed were sampled for DNA extraction. Blood samples were collected from the wing veins of domestic geese breeds. In addition, 21 male L, 21 male S, 22 male W and 304 Tianfu meat geese (60 male ; 244 female) breed were sampled for mutation loci validation experiments. The Tianfu meat geese breed was a composite of 87.5 % Landes (*A. anser*) and 12.5 % of Sichuan White geese (*A. cygnoides*). The population was closed and has been artificially selected for 10 generations.

The S used for histomorphological and transcriptomic analysis were provided by Baisha Livestock and Poultry Original Species Research Institute (Shantou, Guangdong). The W and L used for histomorphological and transcriptomic analysis and the population of Tianfu meat geese breed were provided by the Waterfowl Breeding Experimental Farm of Sichuan Agricultural University (Ya’an, Sichuan). All of these geese were provided with free access to feed and water under natural light and temperature condition. These geese were euthanized by inhaling carbon dioxide and cervical dislocation, which performed by competent personnel who experienced and correctly applied the technique. As shown in Additional file 2: Figure S1, both S and W were characterized by a protruding knob at the base of the upper bill and were thus defined as the knob geese; in contrast, L was devoid of the knob and was defined as the non-knob geese. For knob geese, both the skin and bone located in the knob were isolated, while for non-knob group, the skin and adjacent bone in an inverted triangle at the base of the upper bill were collected. The skin and bone from two groups were fixed with 4 % paraformaldehyde for sections or were frozen in liquid nitrogen until RNA extraction.

### Radiograph

Heads of L and W were right latero-lateral radiographed using a detector (MNC 101, MEDN + VA Co., Ltd, Hangzhou, China) with an output of 70 kV and 125 mA in Animal Hospital of Sichuan Agricultural University in Ya’an, China. Similarly, heads of S were right latero-lateral radiographed using a detector (PP14”*17”, Sichuan Yi’an Ray Protection Equipment Co., Ltd, Neijiang, China) with an output of 160 V and 220 mA in RuiTeng Pet Hospital in Shantou, Guangdong.

### Histological observation

Both the skin and bone located in the knob of six individuals from each of S, W and L were subjected to histological analysis. In detail, each tissue was paraffin-embedded and then sliced to a thickness of 4 μm. Paraffin sections of the skin and bone were stained with hematoxylin-eosin (H&E). Paraffin sections of the skin were also subjected to Masson staining. All of these paraffin sections were photographed under a Nikon 90i microscope (Nikon, Japan) and scanned by a digital pathological section scanner (Pannoramic MIDI, 3D Histech, Hungary). The histological parameters of skin were determined according to the standards of healthy human skin in different body regions [46].

### RNA-seq library preparation, sequencing and analysis

Both the skin and bone located in the knob of three individuals from each of S, W and L were used for construction of RNA-seq libraries. Total RNA was extracted from the skin and bone using Trizol reagent (Ambion, USA) following the manufacturer’s instructions. A total of eighteen cDNA libraries were generated using NEBNext® UltraTM RNA Library Prep Kit for Illumina® (NEB, USA) following manufacturer’s recommendations. After generation, the libraries were sequenced on an Illimina Novaseq platform and 150 bp paired-end reads were generated.

The clean data were obtained by removing reads containing adapter, reads containing poly-N and low-quality reads from raw data. At the same time, Q20, Q30 and GC content of the clean data were calculated. The clean data were aligned to the *Anser cygnoides* genome (https://www.ncbi.nlm.nih.gov/assembly/GCF_000971095.1/) using Hisat2 v2.0.5 [47]. Principal component analysis (PCA) of these libraries was created by ggplot2 software package in R (v3.6.1) using custom script. Expression levels of genes were quantified as the expected number of Fragments Per Kilobase of transcript sequence per Millions (FPKM) base pairs sequenced [48]. Differentially expressed genes (DEGs) between pairwise comparisons were identified using the DESeq2 R package (1.16.1) [49], under the criteria of |log_2_ fold change (FC)| ≥ 1 and *p*- adjusted value < 0.05. To obtain DEGs of the knob skin between knob- and non-knob geese, the DEGs in pairwise comparisons (skin of S versus skin of L (SP vs. LP)), skin of W versus skin of L (WP vs. LP)) were intersected. Similarly, to obtain DEGs of the knob bone between knob- and non-knob geese, the DEGs in pairwise comparisons (bone of S versus bone of L (SG vs. LG), bone of W versus bone of L (WG vs. LG)) were also intersected. The Kyoto Encyclopedia of Genes and Genomes (KEGG) enrichment analyses of DEGs were performed by KOBAS 3.0 (http://kobas.cbi.pku.edu.cn/kobas3/genelist/) based on *Gallus gallus* database.

### Whole genome re-sequencing analysis

Genomic DNA extracted from the blood of 3 domestic geese breeds (n = 6 per breed) was used to construct libraries with ~ 350 bp paired-end reads for the whole genome re-sequencing. The re-sequencing data were mapped to the *Anser Cygnoides* reference genome (https://www.ncbi.nlm.nih.gov/assembly/GCF_000971095.1/) with Burrows-Wheeler alignment (BWA aln, v.0.7.8) [50] using default parameters. Genome Analysis Toolkit (GATK) v.3.7 [51] was used to detect variations using custom script. Population structure analysis was performed based on single nucleotide polymorphisms (SNPs) by GCTA v.1.24.2 [52] and R (v3.6.1) using custom script. The selective sweep screen was performed by searching the genome for regions with high fixation index (*F*st) values and high differences in genetic diversity (log2(θ_π_ ratio)) using sliding 50-kb windows with 10-kb steps along the autosomes using VCFtools (version 0.1.14) [53] and inhouse scripts. To detect characteristics that have undergone selection associated with the knob phenotype, genetic variants were further identified in all examined individuals. The genome selection signaling with significantly high *Fst* (corresponding to the top 5 % level) and *θπ* ratio (*θ*_*π*, non−knob geese_*/θ*_*π*, knob geese_, top 5 % level presented) values were identified as extensively diversified by custom scripts. ANNOVAR [54] was used to annotate the selected genes in genome selection signaling with high *Fst* and *θπ* ratio.

### Integrative analysis of RNA-Seq and whole genome re-sequencing data

To identify crucial candidate genes associated with the knob phenotype in goose, candidate genes under positive selection (PSGs) were intersected with DEGs of skin and bone located in the knob between knob- and non-knob geese by VENNY 2.1.0 (https://bioinfogp.cnb.csic.es/tools/venny/index.html). Thereafter, the analysis of the alleles’ frequency difference between the knob- and non-knob geese was realized by R and Perl, and the variations located in the coding region of candidate genes were identified by hand. The protein sequences of candidate genes in various species were downloaded from NCBI (https://www.ncbi.nlm.nih.gov/protein/), the sequence accessions were listed in Additional file 1: Table S2. DNAMAN software was used to compare the homology and create the phylogenetic relationship for candidate genes among various species. Functional domains of candidate genes were predicted by online CDD-Search in NCBI. ExPASy.ProtParam [55] was used to predict the physicochemical properties of candidate genes. The secondary structures of candidate genes were predicted by SOPMA software [56]. The mutation loci of candidate genes were validated by Sanger sequencing in the population of S, W and L as well as the population of Tianfu meat geese breed. The primers used for validation were listed in Additional file 1: Table S1. The PCR reaction system was performed in a total volume of 20 µl using 10 µl 2 x PCR HeroTM Mix(dye) (FOREGENE, Chengdu, China), 2 µl DNA, 0.4 µl Primers (10 µM each) and 7.2 µl ddH_2_O. The PCR conditions were 94 ℃ pre-denaturation for 3 min, 35 cycles at 94 ℃ denaturation for 10 s, annealing temperature for 10 s, 72 ℃ extension 20 s, with a final extension at 72 ℃ for 5 min. The PCR products were examined by 1.5 % agarose gel and then were sequenced by Qinke Gene Biotechnology Co. Ltd (Chengdu, China).

### Quantitative real-time PCR (qRT-PCR)

Expression of mRNAs in skin and bone located in the knob was verified by qRT-PCR from the same batch of samples used for RNA-sEq. Total RNA was extracted using Trizol regent (Ambion, USA) following the manufacturer’s instructions. Approximately 1 µg of total RNA was used to synthesize complementary DNA (cDNA) using a cDNA synthesis kit (Takara, China). QRT-PCR was performed on a CFX96TM Real-Time System (Bio-Rad, CA, USA) using SYBR PrimerScriptTM RT-PCR kit (Takara, China). The reactions were as follows: pre-denaturation at 95 ℃ for 5 min, followed by 40 cycles of denaturation at 95 ℃ for 30 s and annealing at corresponding temperature of each primer set for 30 s, extension at 72 ℃ for 30 s. Target specificity for each primer set was validated by melting curve analyses. The expression of mRNAs was evaluated by 2^(Cq(reference)−Cq(target))^ [57]. Geese *GAPDH* and *β-actin* were normalized as housekeeping genes. Each sample was in triplicate. Primers designed for qRT-PCR were listed in Additional file 1: Table S1.

### Data analysis

Image J software [58] was used to calculate the degree of the bone calcification located in the knob of S, W and L. CaseViewer software was used to measure the histological parameters of sections of skin and bone located in the knob. All data were sorted out by Excel 2019 for further analysis. Data including the degree of the bone calcification, the histological parameters of the knob skin and bone, and the relative expressions of genes were analyzed to ANOVA using SPSS (version 23.0). The data of non-knob group (L) was used for normalization of the data in knob group (S and W), and the data was presented as multiples of changes. *P* < 0.05 was considered to be statistically significant. The results of Sanger sequencing were viewed by BioEdit software (https://bitesizebio.com/10238/bioedit-a-sequence-alignment-editor-and-it-is-free/) to ensure the mutation loci. The information of allele and genotype of candidate gene was sorted by Excle 2019. Allele and genotype frequencies were determined by direct counting. In addition, the expressions of candidate genes which validated by qRT-qPCR were clustered by Mev 4.9 [59].

## Supplementary Information

**Additional file 1: Table S1.** The primers for qRT-PCR and SNP of candidate genes. **Table S2.** The sequence accessions of DIO2 protein in various species. **Table S3.** Summary of RNA-seq data for each sample. **Table S4.** Mapping statistics for each sample for transcriptom. **Table S5.** The number of differentially expressed genes. **Table S6.** Mapping statistics for each individual for the whole genome re-sequencing. **Table S7.** SNP calling for the whole genome re-sequencing. **Table S8.** Positively selected genes harbored in the positively selected regions. **Table S9.** The expression pattern and population differentiation values of candidate genes.

**Additional file 2: Figure S1.** The morphology of different knob phenotype. (A) S, Lion head goose, (B) W, Sichuan White goose, (C) L, Landes goose. Knob is circled in knob goose, and the position of knob in non-knob goose is indicated using arrow. **Figure S2.** Histological parameters of the knob skin in three domestic geese breeds. Different letters indicate significant differences among different breeds at *P* < 0.05. S, Lion head goose; W, Sichuan White goose; L, Landes goose. **Figure S3. **Principal component analysis (PCA) of the knob skin and bone in three geese breeds, respectively. (A) Skin located on knob. (B) Bone located in knob. SP, skin located on knob in Lion head goose; WP, skin located on knob in Sichuan White goose; LP, skin located on knob in Landes goose; SG, bone located in knob in Lion head goose; WG, bone located in knob in Sichuan White goose; LG, bone located in knob in Landes goose. **Figure S4. **qRT-PCR validation of expression of DEGs and PSGs. The qRT-PCR data of knob geese (S and W) were normalized by that of non-knob geese (L) and signified by dashed line, while the RNA-seq data were signified by solid line. SP, skin located on knob in Lion head goose; WP, skin located on knob in Sichuan White goose; LP, skin located on knob in Landes goose; SG, bone located in knob in Lion head goose; WG, bone located in knob in Sichuan White goose; LG, bone located in knob in Landes goose. DEGs, differentially expressed genes; PSGs, candidate genes under positive selection.

## Data Availability

All data generated or analyzed during this study are included in this published article and its additional files, or in the following public repositories. Data has been submitted to the public database under the following accession numbers: whole genome re-sequence data [PRJNA671609] (http://www.ncbi.nlm.nih.gov/bioproject/671609); transcriptome sequence data [PRJNA669912] (http://www.ncbi.nlm.nih.gov/bioproject/669912).

## Abbreviations

DEGs: Differentially expressed genes
PSGs: Candidate genes under positive selection
L: Landes goose
S: Lion head goose
W: Sichuan White goose
H&E: Hematoxylin-eosin
PCA: Principal component analysis
FPKM: Fragments Per Kilobase of transcript sequence per Millions
FC: Fold change
SP: Skin of Lion head goose
LP: Skin of Landes goose
WP: Skin of Sichuan white goose
SG: Bone of Lion head goose
LG: Bone of Landes goose
WG: Bone of Sichuan white goose
KEGG: Kyoto Encyclopedia of Genes and Genomes
GATK: Genome Analysis Toolkit
SNPs: Single nucleotide polymorphisms
*F*st: The population differentiation statistic
*θ*_*π*_: The sequence diversity statistic
qRT-PCR: Quantitative real-time PCR
SC: Stratum corneum
SG: Stratum granulosum
SS: Stratum spinosum
SB: Stratum basal
CAMs: Cell adhesion molecules
*RXFP2*: Relaxin family peptide receptor 2
*DIO2*: Iodothyronine deiodinase 2
*KIAA2022*: Neurite extension and migration factor
*ADAMTS3*: ADAM metallopeptidase with thrombospondin type 1 motif 3
TRPC6: Transient receptor potential cation channel subfamily C member 6
*EPHX4*: Epoxide hydrolase 4
*GSC*: Goosecoid homeobox
*TVP23A*: Trans-golgi network vesicle protein 23 homolog A
*CIITA*: Class II major histocompatibility complex transactivator
*FBXL17*: F-box and leucine rich repeat protein 17
*RFWD2*: COP1 E3 ubiquitin ligase
*CACNA1I*: Calcium voltage-gated channel subunit alpha1 I
*TENM1*: Teneurin transmembrane protein 1
*FBN2*: Fibrillin 2
*RGS7*: Regulator of G protein signaling 7
*RIMS1*: Regulating synaptic membrane exocytosis 1
*PLA2G4F*: Phospholipase A2 group IVF
*ABI3BP*: ABI family member 3 binding protein
*HMMR*: Hyaluronan mediated motility receptor
*PRLR*: Prolactin receptor
*LPL*: Lipoprotein lipase
*VTN*: Vitronectin
*THRB*: Thyroid hormone receptor beta
*ADCY2/8*: Adenylate cyclase 2/8
*NOS2*: Nitric oxide synthase 2
*PLCB1*: Phospholipase C beta 1
*FABP3/7*: Fatty acid binding protein 3/7
*GRIN1*: Glutamate ionotropic receptor NMDA type subunit 1
*CAMK1G*: Calcium/calmodulin dependent protein kinase IG
ECM: Extracellular matrix
*PPARγ*: Peroxisome proliferator activated receptor gamma
ERK1/2: Extracellular signal-regulated kinase1/2
T4: Thyroxine
T3: Triiodothyronine
THs: Thyroid hormones
TSH: Thyroid stimulating hormone
