# Supplementary material for: Integrative analysis of histomorphology, transcriptome and whole genome resequencing identified DIO2 gene as a crucial gene for the protuberant knob located on forehead in geese
Source: BMC Genomics. 2021 Jun 30;22:487. doi: 10.1186/s12864-021-07822-9 (PMC8244220; doi:10.1186/s12864-021-07822-9)
Supplement: Supplementary file 2 — Additional file 2: Figure S1. The morphology of different knob phenotype. (A) S, Lion head goose, (B) W, Sichuan White goose, (C) L, Landes goose. Knob is circled in knob goose, and the position of knob in non-knob goose is indicated using arrow. Figure S2. Histological parameters of the knob skin in three domestic geese breeds. Different letters indicate significant differences among different breeds at P < 0.05. S, Lion head goose; W, Sichuan White goose; L, Landes goose. Figure S3. Principal component analysis (PCA) of the knob skin and bone in three geese breeds, respectively. (A) Skin located on knob. (B) Bone located in knob. SP, skin located on knob in Lion head goose; WP, skin located on knob in Sichuan White goose; LP, skin located on knob in Landes goose; SG, bone located in knob in Lion head goose; WG, bone located in knob in Sichuan White goose; LG, bone located in knob in Landes goose. Figure S4. qRT-PCR validation of expression of DEGs and PSGs. The qRT-PCR data of knob geese (S and W) were normalized by that of non-knob geese (L) and signified by dashed line, while the RNA-seq data were signified by solid line. SP, skin located on knob in Lion head goose; WP, skin located on knob in Sichuan White goose; LP, skin located on knob in Landes goose; SG, bone located in knob in Lion head goose; WG, bone located in knob in Sichuan White goose; LG, bone located in knob in Landes goose. DEGs, differentially expressed genes; PSGs, candidate genes under positive selection. [file 12864_2021_7822_MOESM2_ESM.docx]

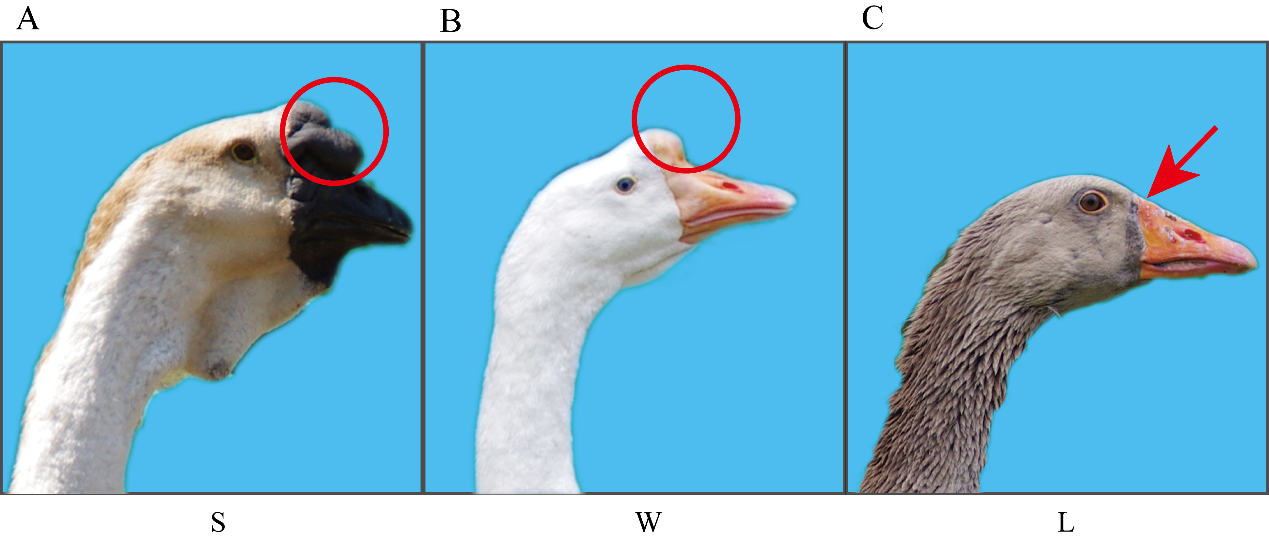


**Additional file 2 Figure S1.**


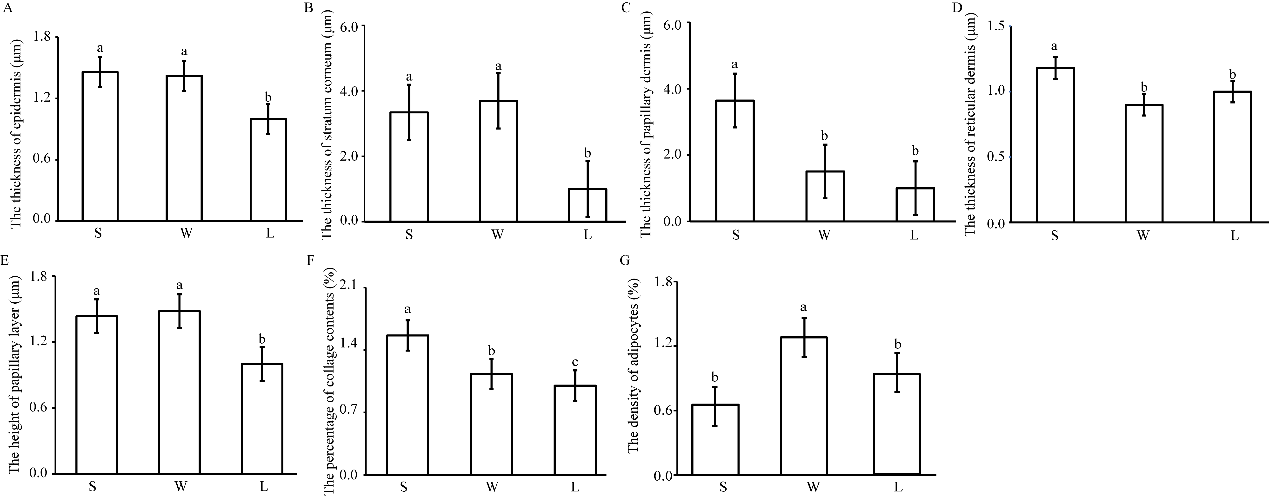


**Additional file 2 Figure S2.**


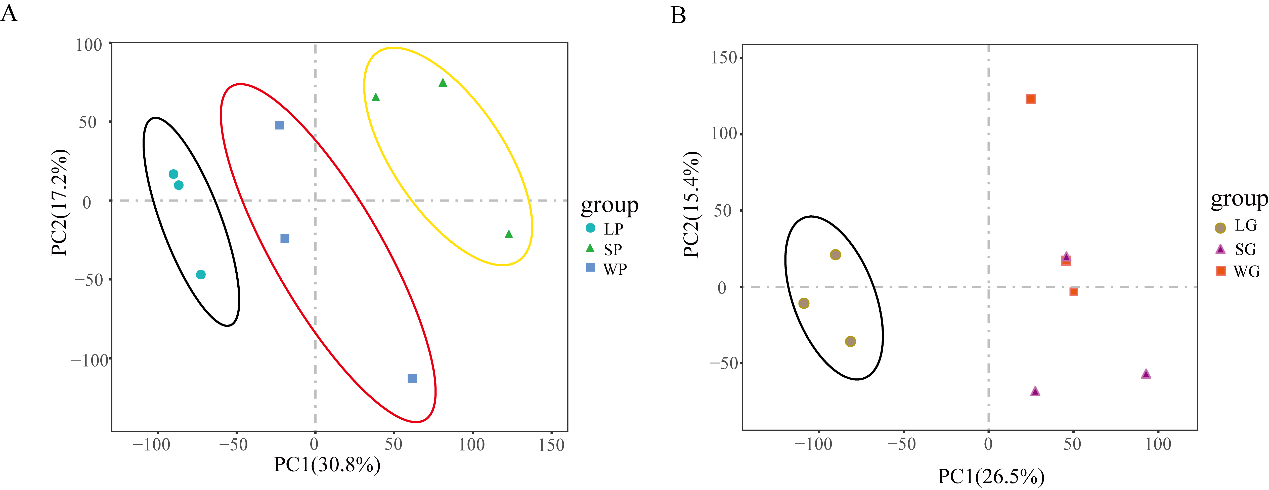


**Additional file 2 Figure S3.**


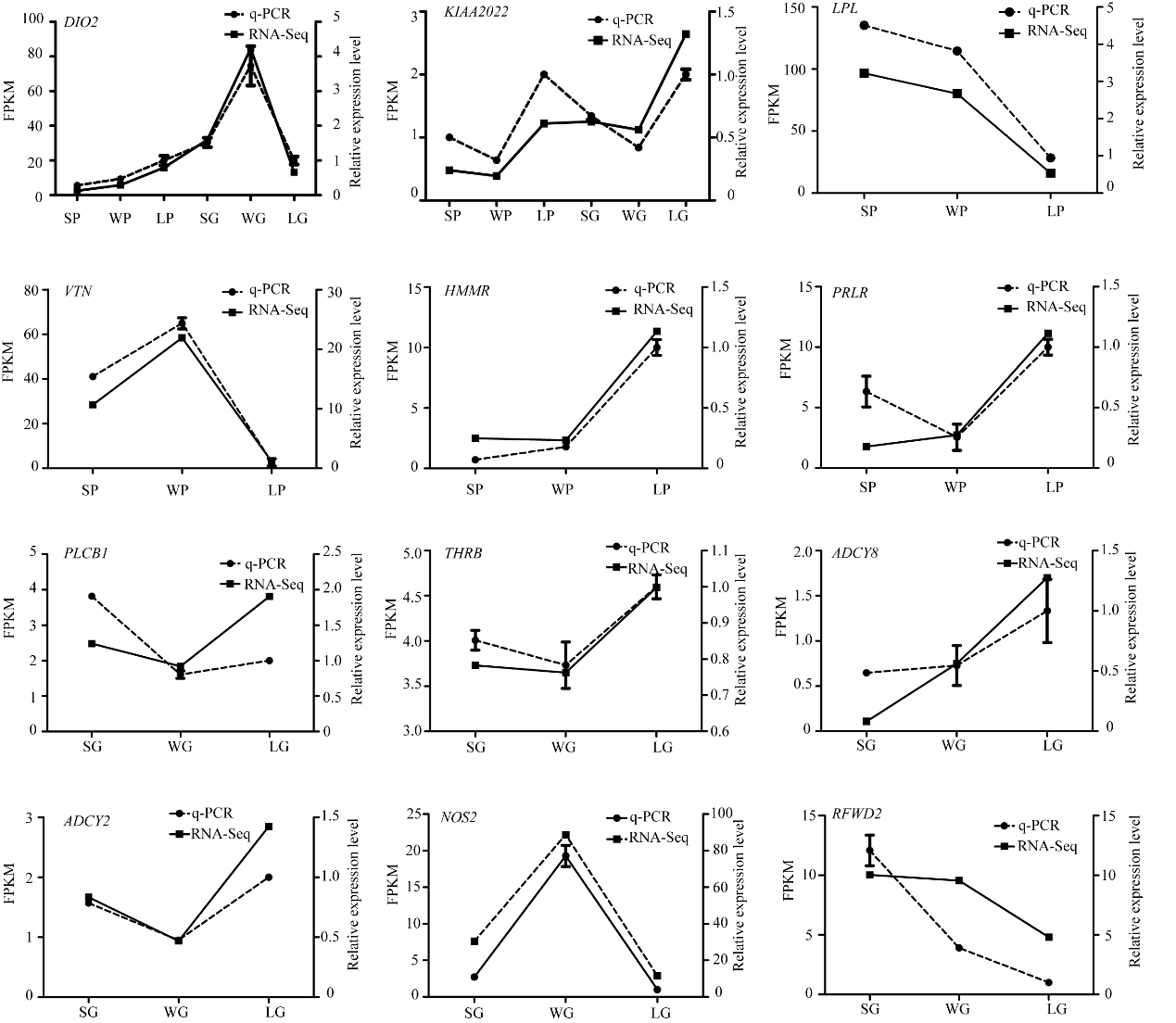


**Additional file 2 Figure S4.**
